# Species delimitation in lemurs: multiple genetic loci reveal low levels of species diversity in the genus *Cheirogaleus*

**DOI:** 10.1186/1471-2148-9-30

**Published:** 2009-02-04

**Authors:** Linn F Groeneveld, David W Weisrock, Rodin M Rasoloarison, Anne D Yoder, Peter M Kappeler

**Affiliations:** 1 Sociobiology and Behavioral Ecology, German Primate Center, Kellnerweg 4, 37077 Göttingen, Germany; 2 Department of Biology, Duke University, Durham, NC, USA; 3 Département de Biologie Animale, Université d'Antananarivo, Antananarivo, Madagascar; 4 Sociobiology and Anthropology, Institute for Zoology, University of Göttingen, Göttingen, Germany; 5 Breeding and Genetic Resources, Institute of Farm Animal Genetics, Friedrich Loeffler Institut, 31535 Neustadt-Mariensee, Germany; 6 Department of Biology, University of Kentucky, Lexington, KY, USA

## Abstract

**Background:**

Species are viewed as the fundamental unit in most subdisciplines of biology. To conservationists this unit represents the currency for global biodiversity assessments. Even though Madagascar belongs to one of the top eight biodiversity hotspots of the world, the taxonomy of its charismatic lemuriform primates is not stable. Within the last 25 years, the number of described lemur species has more than doubled, with many newly described species identified among the nocturnal and small-bodied cheirogaleids. Here, we characterize the diversity of the dwarf lemurs (genus *Cheirogaleus*) and assess the status of the seven described species, based on phylogenetic and population genetic analysis of mtDNA (*cytb *+ *cox2*) and three nuclear markers (*adora3*, *fiba *and *vWF*).

**Results:**

This study identified three distinct evolutionary lineages within the genus *Cheirogaleus*. Population genetic cluster analyses revealed a further layer of population divergence with six distinct genotypic clusters.

**Conclusion:**

Based on the general metapopulation lineage concept and multiple concordant data sets, we identify three exclusive groups of dwarf lemur populations that correspond to three of the seven named species: *C. major*, *C. medius *and *C. crossleyi*. These three species were found to be genealogically exclusive in both mtDNA and nDNA loci and are morphologically distinguishable. The molecular and morphometric data indicate that *C. adipicaudatus *and *C. ravus *are synonymous with *C. medius *and *C. major*, respectively. *Cheirogaleus sibreei *falls into the *C. medius *mtDNA clade, but in morphological analyses the membership is not clearly resolved. We do not have sufficient data to assess the status of *C. minusculus*. Although additional patterns of population differentiation are evident, there are no clear subdivisions that would warrant additional specific status. We propose that ecological and more geographic data should be collected to confirm these results.

## Background

In most biodiversity and conservation assessments species are the fundamental unit by which diversity is measured (e.g [1-3]). Depending on the criteria used to recognize species, vastly different numbers and distributions can be identified. For example, the difference in species numbers when utilizing a phylogenetic species concept (e.g. [4,5]) versus a biological species concept (e.g. [6]) can be substantial. Agapow et al. [7] estimated a 48% increase in recognized species across a wide range of organisms (ranging from fungi to mammals) when using a phylogenetic species concept. Similarly, Zink [8] proposed a doubling of known bird species, mostly due to the elevation of subspecies to full specific status. Such a drastic difference in species numbers would necessitate an extensive revision of most conservation measures. Furthermore species are the fundamental unit of comparison in all subdisciplines within biology (e.g. [9]). As such, robust measures of species delimitation and boundaries are crucial to understanding the evolution of organisms and how best to manage biodiversity in the face of increased anthropogenic pressure.

The lemuriform primates of Madagascar have undergone a recent explosion in species descriptions, with as many as 47 new species described in the last 25 years as a result of intensified field work, the incorporation of molecular data in the identification of previously cryptic species, and a paradigm shift in what we recognize as a species [10-12]. This increase has come in the face of tremendous anthropogenic pressures, with Madagascar having just a fraction of its original native habitat remaining [13]. Increased recognition of lemuriform species diversity has been particularly acute in the family Cheirogaleidae, a clade of small-bodied and nocturnal lemurs with a generally cryptic morphology. In just over 10 years the number of recognized cheirogaleid species has more than quadrupled with most of this activity occurring in mouse lemurs of the genus *Microcebus *(e.g. [14-20]).

The dwarf lemur genus *Cheirogaleus *has received considerably less taxonomic attention despite having an island-wide distribution and sharing similar habitats with mouse lemurs. This genus consisted of two species from the 1930s until the turn of the last century, with only the number of recognized subspecies varying between authors. It was proposed that a grayish colored species, *Cheirogaleus medius*, inhabited the western dry forests, and a larger rufus-colored form, *Cheirogaleus major*, occupied the eastern rainforests [21-23]. Using descriptive morphological assessments of existing museum material, Groves [24] split *C. medius *into two species: (1) *C. medius *in western Madagascar and (2) *C. adipicaudatus *in the south. *Cheirogaleus major *was split into five species: (1) *C. major *with a broad eastern distribution, (2) *C. crossleyi*, which is found more inland than *C. major *and also extends further north, (3) *C. minusculus*, known only from a single eastern locality at Ambositra, (4) *C. ravus*, which has a narrow coastal range within *C. major*, and (5) *C. sibreei*, with an unclear distribution, but known from an eastern locality at Ankeramadinika and possibly from the northwest at the Ampasindava Bay. While maintaining these two groups (*medius *and *major*), Groves [24] noted that he did so only for convenience. Nonetheless, he found these seven taxa to represent distinguishable morphs and interpreted them as separate genetic entities. However, until now, no comprehensive study of geographic and genetic variation has been performed to test the hypothesis that these seven taxa represent distinct phylogenetic species.

Only one study has assessed the geographic patterning of genetic variation in *Cheirogaleus*. Hapke et al. [25] used mitochondrial DNA (mtDNA) sequence data in an attempt to clarify the species status of three different morphotypes resembling *C. crossleyi*, *C. major*, and *C. medius *found in close proximity in the Fort Dauphin area of southeastern Madagascar. Using dense sampling in this area along with representatives of *C. crossleyi*, *C. major*, and *C. medius *from other portions of the island, Hapke et al. [25] resolved three mtDNA haplotype clades each exclusive to one of the three representative species. These results are the only genetic evidence to date that some of the species described by Groves [25] represent independent lineages. They also greatly expand the potential range of *C. crossleyi *into the southern portion of the island. Evidence for the exclusivity of *C. adipicaudatus, C. minusculus, C. ravus*, and *C. sibreei *is still lacking.

Robust studies of species delimitation should take into account both geographic and genetic variation in the recognition of species-level lineages. Field sampling of individuals should be sufficient to characterize the frequency of alleles within a single locality and also sufficient to characterize their spatial distribution [26]. Genetic sampling should be sufficient to provide some understanding of the genealogical variation that exists across independent loci as a result of the lineage sorting process and gene flow [27,28]. Species delimitation approaches that take the above into account and search for concordant patterns across independent data sets have been proposed and employed by numerous researchers in the past [e.g. [27,29-34]]. However, recent efforts in lemur species delimitation have raised concerns regarding the methods and data used for the diagnosis of species-level lineages [12]. The majority of recent descriptions has relied primarily on mtDNA, using either genetic distances or fixed substitutions as criteria for species recognition or has not provided proper holotypes [e.g. [18-20]]. These practices beg the question whether such data and their analysis are sufficient to reliably diagnose species-level units, despite the potential for gene tree-species tree discordance due to gene flow or lineage sorting [28,35,36].

In this study we aim to provide a more comprehensive assessment of species diversity in the genus *Cheirogaleus *using an expanded geographic and genetic sampling approach. We use a concordance-based approach [see e.g. [27,29,33]] across independent sources of mitochondrial and nuclear DNA sequence data to identify independently evolving lineages according to the General Lineage Concept of species [9,37]. We also explore a finer level of resolution using population-genetic structuring methods to diagnose sets of populations that are genetically distinct and, which may represent more recently diverged, but independently evolving population-level lineages.

In our molecular analyses we included field samples, museum samples and already published sequences from GenBank. With this multilocus data set and with references to recently collected morphological data, we aim to provide the best estimate of diversity in the genus *Cheirogaleus *currently possible and test the exclusivity of the seven recognized species. If we view taxonomic classifications as scientific hypotheses that may be refined and revised with new data [38,39], our study can contribute significantly towards clarification and interpretation of dwarf lemur diversity.

## Results

### Haplotype data

**Figure 1:**
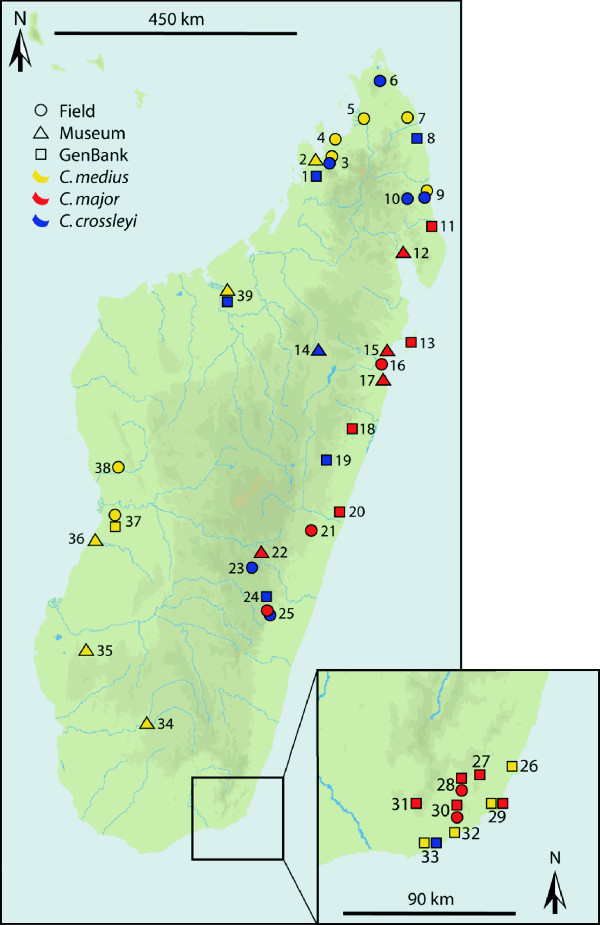
**Sampling localities used in this study**. Field samples collected by the authors are marked with circles. Presumed sites of origin for museum specimens are marked by triangles. Localities for GenBank samples are marked by squares. Symbols are colored according to the three main clades defined in the mtDNA gene tree (Fig. 2). More than one symbol can refer to one locality, if multiple species, or multiple types of data are found at one site. Detailed information for locality sites, marked by locality number, are given in Tables 1-3 and in Hapke et al. [25].

The concatenated *cytb *and *cox2 *sequences from the 48 field samples (Table 1) amounted to 1824 bp and contained no indels. There were 468 variable sites defining 29 haplotypes. A fragment of 246 bp was obtained from museum samples of 16 individuals (Table 2). Among these 16 samples, there were 48 variable sites defining 9 haplotypes. 24 *Cheirogaleus *haplotypes from GenBank, consisting of 17 complete and five partial (307–933 bp) *cytb *sequences, and two complete and one partial (529 bp) *cox2 *sequences (Table 3), were aligned with the field and museum haplotypes resulting in an overall set of 62 haplotypes defined through 494 variable sites (Table 4; all sampling sites are given in Fig. 1).

**Table 1:** Field samples included in this study

Unique identifier	Locality	Latitude	Longitude	Locality #
E1001	Ambanja/Ambato	-13.39583	48.47051	4
E1002	Kirindy	-20.07370	44.67567	37
E1003	Kirindy	-20.07222	44.67468	37
E1004	Kirindy	-20.07222	44.67468	37
E1055	Bekaraoka	-13.10470	49.70740	7
RMR132	Marolambo	-20.06022	48.18330	21
RMR133	Marolambo	-20.06022	48.18330	21
RMR134	Marolambo	-20.06022	48.18330	21
RMR135	Marolambo	-20.06022	48.18330	21
RMR137	Marolambo	-20.06022	48.18330	21
RMR139	Tampolo	-17.28683	49.40877	16
RMR140	Tampolo	-17.28683	49.40877	16
RMR141	Tampolo	-17.28683	49.40877	16
RMR146	Andrambovato/Oranjatsy	-21.49593	47.40180	25
RMR148	Andrambovato/Ambalavero	-21.49645	47.44537	25
RMR149	Andrambovato/Ambalavero	-21.49645	47.44537	25
RMR150	Bemaraha	-19.10358	44.76747	38
RMR152	Bemaraha	-19.10358	44.76747	38
RMR153	Montagne d'Ambre	-12.47478	49.21845	6
RMR155	Montagne d'Ambre	-12.47478	49.21845	6
RMR158	Montagne d'Ambre	-12.47478	49.21845	6
RMR162	Ambanja/Benavony	-13.71113	48.47992	3
RMR164	Ambanja/Beandroana	-13.70298	48.50455	3
RMR166	Sambava	-14.39940	50.17387	9
RMR167	Sambava	-14.39940	50.17387	9
RMR168	Sambava	-14.39940	50.17387	9
RMR169	Sambava	-14.39940	50.17387	9
RMR170	Sambava	-14.39940	50.17387	9
RMR171	Sambava	-14.39940	50.17387	9
RMR172	Sambava	-14.39940	50.17387	9
RMR173	Sambava	-14.39940	50.17387	9
RMR174	Sambava	-14.39940	50.17387	9
RMR175	Sambava	-14.39940	50.17387	9
RMR176	Sambava	-14.39940	50.17387	9
RMR177	Sambava	-14.39940	50.17387	9
RMR178	Sambava	-14.39940	50.17387	9
RMR179	Manantenina	-14.49100	49.81145	10
RMR180	Manantenina	-14.49100	49.81145	10
RMR181	Manantenina	-14.47548	49.83905	10
RMR182	Manantenina	-14.47548	49.83905	10
RMR183	Manantenina	-14.47548	49.83905	10
RMR184	Manantenina	-14.47548	49.83905	10
RMR193	Ankazomivady	-20.77995	47.18198	23
RMR194	Ankazomivady	-20.77995	47.18198	23
RMR196	Ankazomivady	-20.77995	47.18198	23
RMR201	Ivorona	-24.82367	46.94870	28
RMR205	Ivorona	-24.82367	46.94870	28
RMR212	Manantantely	-24.98815	46.92212	30

Unique identifier, sampling locality, coordinates in decimal degrees, and number of sampling locality as marked on the map in Fig. 1 are given.

**Table 2:** Museum samples included in this study

Museum	Catalogue number	Species	Unique identifier	Locality	Locality #
MNHN	CG 1932–3364	*C. adipicaudatus*	Mu1045	170 km East of Tulear	34
MNHN	CG 1932–3365	*C. adipicaudatus*	Mu1032*	170 km East of Tulear	34
MNHN	CG 1932–3365	*C. adipicaudatus*	Mu1046	170 km East of Tulear	34
MNHN	CG 1967-1655	*C. medius*	Mu1042	Ampijoroa	39
MNHN	CG 1932–3362	*C. major*	Mu1044	Maroantsetra	12
MNHN	CG 1964–72	*C. ravus*	Mu1034	Mahambo	17
MNHN	CG 1964–74	*C. ravus*	Mu1033	Ambodivoangy	-
Naturalis	1887:66b	*C. sibreei*	Mu1014	Baie de Passandava	2
Naturalis	D.C. van Dam e	*C. medius*	Mu1020	Mouroundava	36
Naturalis	D.C. van Dam a	*C. medius*	Mu1015	Mouroundava	36
Naturalis	1887:66f	*C. major*	Mu1022	Passumbée	15
Naturalis	1887:66g	*C. major*	Mu1011	Maranzettra	12
Naturalis	1887:66c	*C. major*	Mu1012	Madagascar	-
NHM	1948.160	*C. crossleyi*	Mu1050	Lake Alaotra	14
NHM	1935.1.8.168	*C. adipicaudatus*	Mu1051	Tabiky	35
NHM	1939.1289	*C. crossleyi*	Mu1053	Imerina, E.	22
NHM	1935.1.8.169	*C. major*	Mu1054	Maroantsetra	12

Museum the specimen is housed at, catalogue number of the specimen, species label as recorded by the museum (MNHN = Muséum National d'Histoire Naturelle, Naturalis – Nationaal Natuurhistorisch Museum, NHM = Natural History Museum), unique identifier, locality of provenance as indicated by the museum catalogues, and locality number as used in Fig. 1 are given. * same individual as Mu1046.

**Table 3:** GenBank samples included in this study

GBAN	Species	Locality and/or *unique identifier*	Locality #	Locus	Number of bp
AH014105	*C. major*	Nosy Boraha, Ile Ste. Marie	13	*cytb*	208+259+241
AH014106	*C. major*	Mahanoro	20	*cytb*	633+241
AY441457	*C. major*	Andasibe; *JP118*	19	*cytb*	1140
AY584486	*C. medius*	Manongarivo	1	*cox2*	684
AY584487	*C. major*	Ranomafana	24	*cox2*	684
AY605903	*C. medius*	Morondava CFPF	37	*cytb*	1140
AY605904	*C. medius*	Foret de l'Ankarana	5	*cytb*	933
AY605905	*C. medius*	Ste. Luce	26	*cytb*	1140
AY605906	*C. medius*	Ste. Luce	26	*cytb*	1140
AY605907	*C. medius*	Ste. Luce, Mandena	26, 29	*cytb*	1140
AY605908	*C. medius*	Mandena	29	*cytb*	1140
AY605909	*C. medius*	Petriky, Lavasoa	32, 33	*cytb*	1140
AY605910	*C. medius*	Lavasoa	33	*cytb*	1140
AY605911	*C. major*	Maroantsetra	11	*cytb*	1140
AY605915	*C. major*	Toamasina/Tamatave	18	*cytb*	1140
AY605918	*C. major*	Andohavondro	31	*cytb*	1140
AY605919	*C. major*	Manantantely	30	*cytb*	1140
AY605920	*C. major*	Manantantely, Mandena	30, 29	*cytb*	1140
AY605921	*C. major*	Ivorona	28	*cytb*	1140
AY605922	*C. major*	Farafara	27	*cytb*	1140
AY605923	*C. major*	Farafara	27	*cytb*	1140
AY605926	*C. crossleyi*	Iharana/Vohemar	8	*cytb*	633
AY605927	*C. crossleyi*	Lavasoa	33	*cytb*	1140
EF122247*	*C. medius*	Ampijoroa	39	*cox2*	529
EF122249	*C. medius*	Ampijoroa	39	*cytb*	307
AF285543	*Microcebus berthae*	*Jorg46*	-	*cytb*	1140
AF285507	*Microcebus berthae*	*Jorg46*	-	*cox2*	684
AF285530	*Microcebus ravelobensis*	*RMR53*	-	*cytb*	1140
AF285494	*Microcebus ravelobensis*	*RMR53*	-	*cox2*	684
AF285564	*Microcebus murinus*	*RMR24*	-	*cytb*	1140
AF321177	*Microcebus murinus*	*RMR24*	-	*cox2*	684
EF052512	*Microcebus berthae*	*voucher 149*	-	*adora3*	370
DQ003347	*Microcebus berthae*	*voucher 149*	-	*fiba*	605
EF052411	*Microcebus berthae*	*voucher 149*	-	*vWF*	773
EF052561	*Microcebus ravelobensis*	*voucher 66*	-	*adora3*	370
DQ003410	*Microcebus ravelobensis*	*voucher 66*	-	*fiba*	605
EF052462	*Microcebus ravelobensis*	*voucher 66*	-	*vWF*	758
EF052619	*Microcebus murinus*	*voucher 203*	-	*adora3*	370
DQ003447	*Microcebus murinus*	*voucher 203*	-	*fiba*	600
EF052508	*Microcebus murinus*	*voucher 203*	-	*vWF*	703
EU342234	Mirza coquereli**	*DLC2307*	-	*adora3*	370
EU342261	Mirza coquereli**	*DLC2307*	-	*fiba*	603
AY434036	Mirza coquereli**	*DUPC384F*	-	*vWF*	756
U53571	Mirza coquereli**	-	-	*cytb*	1140
AY321460	Mirza coquereli**	*DUPC384F*	-	*cox2*	684

GenBank accession numbers, species label as indicated in GenBank, locality of provenance and or unique identifier of the individual, locality number as used in Fig. 1, locus and number of basepairs available for the respective locus are given. *same individual as EF122249. **listed in GenBank as *M. coquereli*, but have to be reclassified as *Mirza zaza*.

**Table 4:** Data sets and nucleotide substitution models

Data set	Alignment length	# of sequences	# of haplotypes	# of variable/parsimony informative sites	Model ML	Model Bayesian
*cytb *+ *cox2*	1824	88	62	494/442	GTR+I+Γ	GTR+I+Γ
*adora3*	370	96	29	26/17	GTR+I	GTR+I
*fiba*	604	96	49	44/34	K81uf+I+Γ	GTR+I+Γ
*vWF*	793	96	52	93/77	HKY+I+Γ	HKY+I+Γ

The data set, respective alignment length including outgroup, number of sequences in data set excluding outgroup, number of haplotypes excluding outgroup, and number of variable sites excluding outgroup, when gaps are considered missing data, except for the mtDNA data set are given. Nucleotide substitution models for each data set, as used in ML analyses and in Bayesian analyses, are listed.

Nuclear DNA sequence data were generated from the 48 field samples. In all individuals both alleles were scored, amounting to a data set of 96 sequences for each locus. Among *Cheirogaleus *samples, the 370 bp exonic *adora3 *fragment had 26 variable sites and 29 haplotypes. The 604 bp intronic fragment *fiba *had 44 variable sites and 49 haplotypes. The 793 bp intronic fragment *vWF *had 93 variable sites and 52 haplotypes (Table 4). The *adora3 *alignment contained no indels. Both the *fiba *and *vWF *alignments contained a small number of 1–2 bp indels. In addition, the *vWF *alignment contained indels of 19 and 242 bp in six and three individuals, respectively.

The *cytb, cox2*, and *adora3 *loci were each found to best fit a general time-reversible (GTR) model according to AIC. The mtDNA loci were best fit to a model with a proportion of invariant sites (I) and gamma distributed rate heterogeneity (Γ), whereas *adora3 *was best fit to a model with a proportion of invariant sites. A K81uf+I+Γ model was favored for the *fiba *locus, (analyzed under a GTR+I+Γ model in Bayesian phylogenetic analyses). The *vWF *locus was found to best fit an HKY+I+Γ model (Table 4).

### MtDNA gene tree

**Figure 2:**
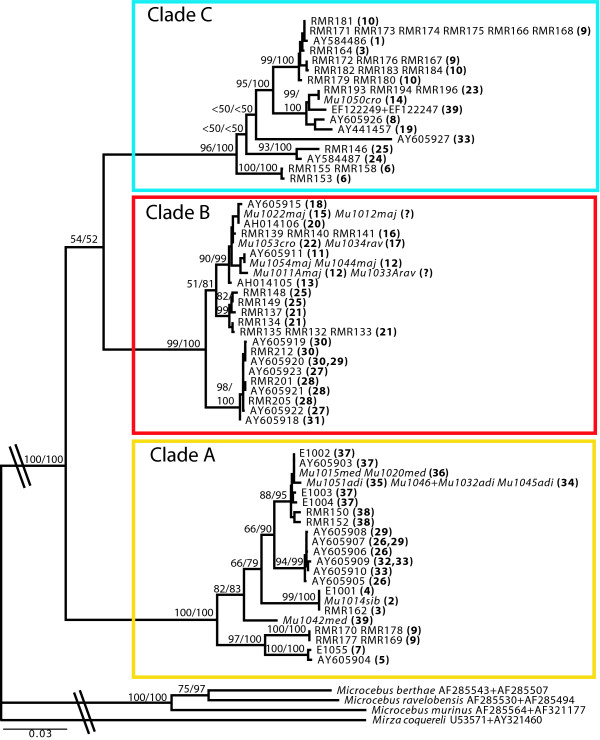
**Maximum likelihood phylogram based on mtDNA**. ML phylogram based on a total alignment of mtDNA *cytb *and *cox2 *haplotype sequences from field and museum samples (in italic) and of published GenBank samples. Tip labels contain the individual field numbers (E, RMR), the museum identifier, or GenBank accession number of sequences within a haplotype. The sampling locality a haplotype was found in, is given in bold type in parentheses, as marked in Fig. 1. GenBank haplotypes may occur in more than one locality. Maximum likelihood bootstrap values and Bayesian posterior probabilities are depicted above the branches.

Bayesian and ML analyses of the mtDNA data set resulted in congruent trees with three main clades (A, B and C in Fig. 2) that largely correspond to the three species recognized prior to the taxonomic revisions of Groves [24]. Clade A is strongly supported (ML BP = 100 and Bayesian PP = 1.0) and consists of haplotypes sampled from western Madagascar, the southeastern tip (Fort Dauphin region) and two sampling sites in the northeast. All mtDNA sequences generated from museum samples of *C. medius*, *C. adipicaudatus*, and *C. sibreei *are placed in clade A.

Clade B is strongly supported (ML BP = 99 and Bayesian PP = 1.0) and is comprised of mtDNA haplotypes sampled from localities along the east coast from the southeastern tip (Fort Dauphin region) to the Maroantsetra peninsula in the northeast (localities 11 and 12). All mtDNA sequences generated from *C. major *and *C. ravus *samples fall into clade B. MtDNA sequence generated from a museum sample of *C. crossleyi *(locality 22) is also placed in Clade B.

Clade C is strongly supported (ML BP = 96 and Bayesian PP = 1.0) and also contains eastern-sampled haplotypes, ranging from the southeastern tip (Fort Dauphin region) up to the northern tip (Montagne d'Ambre, locality 6). Clade C also contains haplotypes sampled from three localities in the northwestern portion of the island (localities 1, 3, and 39). The sole museum-generated sequence placed in Clade C is the *C. crossleyi *individual from locality 14.

The mtDNA-based relationships among clades A, B and C are poorly supported and are best viewed as unresolved. Uncorrected "p" distances based on the *cytb *locus (1140 bp) were calculated for the three main clades (A, B and C). Pairwise distances between the three clades were fairly similar, with an average 11.4% between clades B and C, 12.6% between clades A and B and 13.10% between clades A and C.

### Nuclear gene trees

**Figure 3:**
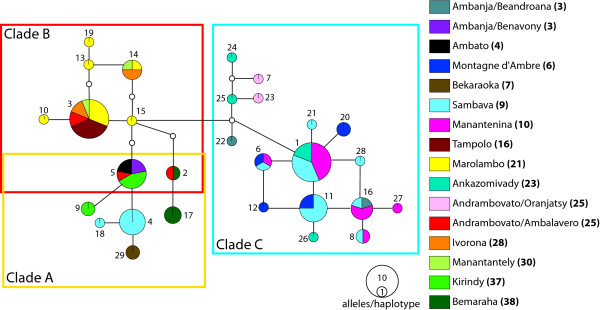
***adora3 *haplotype network**. Statistical parsimony haplotype network representing the genealogical relationships among 29 haplotypes of the *adora3 *locus generated from field-collected samples. Haplotypes are colored according to the respective sampling locality, with the locality number given in the legend in bold as marked in Fig. 1. The sizes of circles representing haplotypes reflect the number of sequences that share a haplotype. Each of the haplotypes is numbered. Inferred intermediate haplotypes, either not sampled, or extinct, are represented by small non-colored circles. Groups of haplotypes found in individuals that correspond to clades A, B and C in the mtDNA tree are outlined by the colored frames.

**Figure 4:**
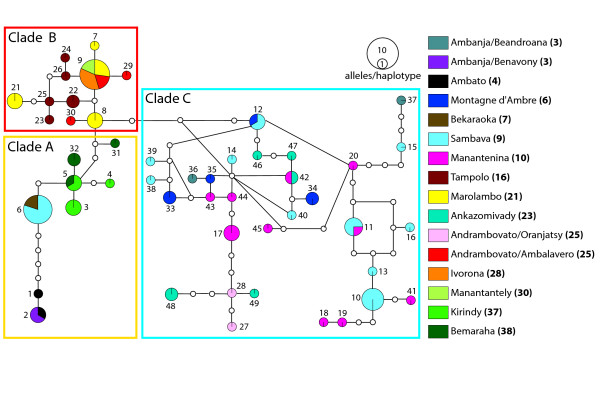
***fiba *haplotype network**. Statistical parsimony haplotype network representing the genealogical relationships among 49 haplotypes of the *fiba *locus generated from field-collected samples. Haplotypes are colored according to the respective sampling locality, with the locality number given in the legend in bold as marked in Fig. 1. The sizes of circles representing haplotypes reflect the number of sequences that share a haplotype. Each haplotype is numbered. Inferred intermediate haplotypes, either not sampled, or extinct, are represented by small non-colored circles. Groups of haplotypes found in individuals that correspond to clades A, B and C in the mtDNA tree are outlined by the colored frames.

**Figure 5:**
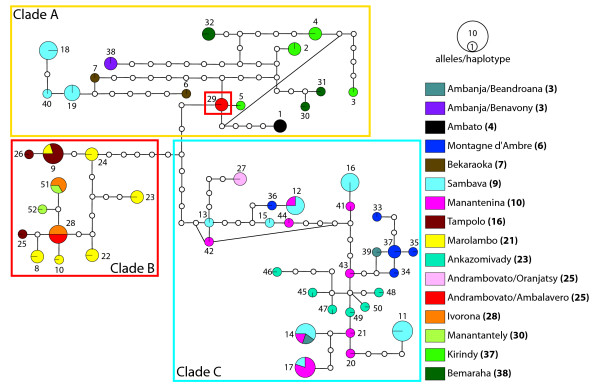
***vWF *haplotype network**. Statistical parsimony haplotype network representing the genealogical relationships among 52 haplotypes of the *vWF *locus generated from field-collected samples. Haplotypes are colored according to the respective sampling locality, with the locality number given in the legend in bold as marked in Fig. 1. The sizes of circles representing haplotypes reflect the number of sequences that share a haplotype. Each haplotype is numbered. Inferred intermediate haplotypes, either not sampled, or extinct, are represented by small non-colored circles. Groups of haplotypes found in individuals that correspond to clades A, B and C in the mtDNA tree are outlined by the colored frames.

Bayesian, ML, and statistical parsimony analyses of the individual nuclear loci resulted in generally congruent gene trees with respect to the resolution of clades A, B, and C identified in the mtDNA gene tree. Bayesian and ML hierarchical nuclear gene trees are presented as figures in additional files 1, 2, 3: *vWF*, *fiba *and *adora3 *ML phylogram. Statistical parsimony haplotype networks are presented in Figs. 3, 4, 5. The *adora3 *haplotype network (Fig. 3) resolves a clade of haplotypes corresponding to clade C in the mtDNA gene tree. The remaining *adora3 *haplotypes collectively correspond to clades A and B in the mtDNA gene tree. Shared polymorphism of *adora3 *haplotypes exists among some individuals assigned to these two mtDNA-based clades (Fig. 3). *Adora3 *haplotype 5 is found in individuals sampled from Ambanja (3), Ambato (4), and Kirindy (37) in the west (all containing Clade A mtDNA haplotypes), and is also sampled from Andrambovato (25) in the east (containing a Clade B mtDNA haplotype). *Adora3 *haplotype 2 is found in individuals sampled from Bemaraha (38) in the west (mtDNA Clade A) and in the Andrambovato locality (mtDNA Clade B).

The *fiba *haplotype network consists of two terminal clades that correspond to clades A and C in the mtDNA gene tree. An internal clade is also resolved corresponding to mtDNA clade B. All haplotypes in this latter clade have a common ancestor in *fiba *haplotype 8 found in a number of individuals sampled from Marolambo (21). These three clades are shallowly diverged from each other. Only two mutational steps separate sampled haplotypes in clades A and B. Only four mutations separate clades B and C.

The *vWF *haplotype network consists of three clades of haplotypes that nearly completely correspond to clades A, B, and C in the mtDNA gene tree. The sole exception to this pattern is individual RMR149 from Andrambovato, which has a clade B mtDNA haplotype, but is homozygous for a "clade A" *vWF *allele.

### Population genetic clustering

**Figure 6:**
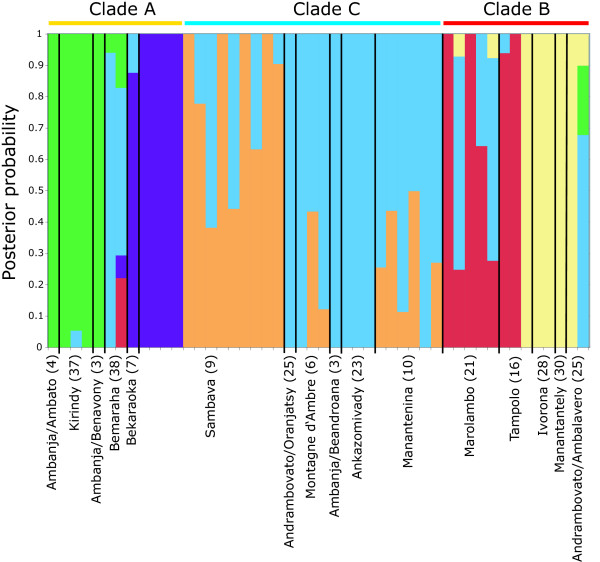
**Bayesian population structure analysis**. Bayesian assignment of the 48 field-collected individuals to populations, based on three nuclear loci, assuming a population number of K = 6. Individuals are arrayed along the x-axis. The y-axis denotes the cumulative posterior probability of an individual's placement in particular population(s). Individuals are divided into sampled populations by thin black lines. Sampled populations are labeled at the bottom with numbers in parentheses corresponding to the sampling locality as marked in Fig. 1.

Bayesian population structure analyses of a combined mtDNA and nuclear data set and a data set comprised of only nuclear loci reveal very similar results (additional file 4: Bayesian population structure analysis), indicating that genetic structuring results are not being driven solely by the mtDNA data. Overall, differences between the results of the two data sets were only found in the number of identical solutions found for each K across replicates, in the exact contribution of each K to the genetic makeup of an individual and in the order that individuals split off to from a separate cluster at K = 4. At K > 6 the number of identical solutions plummets to 0 at a 95% threshold. A K = 6 is the favored solution according to the estimated ln probability of the data (mtDNA + nDNA: average = -1033.2, stdev = 2.4; nDNA: average = -862.0, stdev = 9.5), and according to the ad-hoc statistic ΔK [40], which detected a clear mode at K = 6 for the calculations based on four loci, but showed no clear signal for the three nuclear loci. The K = 6 results from analyses of the combined nuclear data set are described below in the context of the three main mtDNA clades (Fig. 6).

Most individuals possessing clade A mtDNA haplotypes are placed with high PPs in two distinct population clusters (depicted in green and purple in Fig. 6). The genetic compositions of individuals from Ambanja/Ambato (locality 4), Kirindy (37), and Ambanja/Benavony (3) are almost entirely of a single population cluster (green). A subset of individuals from Sambava (9) and the single individual sampled from Bekaraoka (7) are placed almost entirely in a second distinct population cluster (purple). Together with two individuals from Bemaraha (38), which do not fall into either of these two population clusters, the individuals forming these two distinct clusters correspond to clade A mtDNA haplotypes.

All individuals containing clade C mtDNA haplotypes are comprised of two population genetic clusters (depicted in blue and orange in Fig. 6). The remaining Sambava individuals are either completely comprised of, or contain high proportions of, a third population genetic cluster (orange) and low proportions of a fourth population genetic cluster (blue). This pattern is reversed in individuals sampled from Andrambovato/Oranjasty (25), Montagne d'Ambre (6), Ambanja/Beandroana (3), Ankazomivady (23) and Manantenina (10).

Most individuals possessing Clade B mtDNA haplotypes constitute two population genetic clusters (depicted in red and yellow in Fig. 6). All individuals from Marolambo and two individuals from Tampolo are either completely comprised of, or contain some proportion of, a fifth population genetic cluster (red). The individuals sampled from Ivorona, Manantantely, Andrambovato/Ambalavero and the remaining individual from Tampolo, are placed entirely in a sixth cluster (yellow). The sole exception is one of the individuals from Andrambovato/Ambalavero, which is only placed in this cluster with a very low PP.

There is a clear indication that many individuals within mtDNA clades contain a mixed nuclear genetic composition. For example, more than half of individuals with clade C mtDNA haplotypes exhibit a genetic composition from two nuclear-defined clusters (orange and blue).

This pattern also extends across mtDNA-defined clades. For example, some individuals from mtDNA clades A and B can contain a high proportion of a nuclear genetic cluster (blue) that is predominantly found in individuals with clade C mtDNA haplotypes. Overall, these patterns demonstrate the existence of two distinct nuclear genetic clusters within each mtDNA-based clade, but demonstrate the potential for extensive shared genetic makeup within and among these clades.

## Discussion

Groves [24] accepted seven *Cheirogaleus *species, based on morphological data, and interpreted them as separate genetic entities. In this study, mtDNA and nuclear gene sequences clearly resolved only three main lineages within the genus *Cheirogaleus*. Using phylogenetic methods, no further monophyletic subdivisions based on mtDNA and nDNA could be resolved within each of the main lineages. However, a population genetic approach detected a further layer of differentiation, resolving six genetic clusters that largely correspond to, but are not strictly concordant with, the main lineages identified via gene trees. Our results do not rule out the possibility of additional species of dwarf lemurs in Madagascar. There are multiple accounts of sympatric species in areas, which either were not covered by our sampling scheme, or where our sample only contained one morph [41-44]. However, according to our data we can only define three clades that exhibit concordant genealogical patterns across loci.

The three genealogical clades (A, B and C) are largely congruent with three clusters found according to morphometric data (LF Groeneveld, unpublished data). This morphometric data set consisted of data collected from the individuals included in this study and museum specimen and can therefore be directly compared to the genetic data. Species names could be unambiguously assigned to morphometric clusters. *Cheirogaleus medius *and *C. major *differed primarily in size and pelage coloration, while *C. crossleyi *differed from *C. medius *in size and pelage coloration and from *C. major *in pelage coloration and in the width of the skull and in dental characteristics of the premolars and canines (LF Groeneveld, unpublished data) This is congruent with the most recent descriptions of the species [22-24].

Below we discuss the molecular and morphological resolution of evolutionary groups that correspond to population-level lineages and place these within the context of the existing *Cheirogaleus *taxonomy.

### Clade A (*C. medius*)

Clade A is strongly supported in ML and Bayesian analyses of mtDNA sequence data and is also supported by genealogical and population genetic analysis of nuclear data, although shared polymorphism of *adora3 *and *vWF *haplotypes among mtDNA-based clades A and B were detected. These patterns of shared polymorphism may be the result of a low mutation rate, incomplete lineage sorting, or hybridization. Nonetheless, these patterns are limited and do not obscure the concordant patterns of divergence seen across all loci.

The genetically-derived Clade A clearly corresponds to *C. medius *sensu lato according to morphometric and geographic data, as well as the mtDNA sequences generated from *C. medius *museum samples. Analyses of *C. adipicaudatus *museum specimens invariably place individuals into the *C. medius *morphometric cluster and mtDNA clade. These results strongly suggest that *C. adipicaudatus *is synonymous with *C. medius*, with no evidence indicating that it is divergent from other populations of *C. medius*.

The *C. sibreei *individual included in this study is also placed into the *C. medius *clade A. In fact, it shares its mtDNA haplotype with individual RMR162, an individual most likely belonging to *C. medius *according to morphometric characters. These two individuals also cluster together in morphometric analyses (LF Groeneveld, unpublished data). As with *C. adipicaudatus*, these results do not lend support to the hypothesis of *C. sibreei *as a distinct evolutionary group.

### Clade B (*C. major*s)

Clade B is strongly supported in ML and Bayesian analyses of mtDNA and corroborated by nuclear data, although with the patterns of shared polymorphism described above. According to analyses of morphological and mtDNA sequence data generated from museum specimens, clade B corresponds to *C. major*. Analyses of morphological and mtDNA sequence data of *C. ravus *specimens are congruent and place these individuals into the *C. major *morphometric cluster and mtDNA clade B. There is no evidence to suggest that *C. ravus *represents a distinct genetic lineage within the larger *C. major *group and we conclude that *C. ravus *is synonymous with *C. major*. Interestingly, one of the museum-sampled individuals of *C. crossleyi *(Mu1053) contains an mtDNA haplotype that is placed within mtDNA clade B. However, it is difficult to infer anything else about this *C. crossleyi *museum specimen since this is a juvenile individual and was not included in the morphological analyses. Other putatively *C. crossleyi *museum and field-collected individuals are consistently placed outside the *C. major *group, suggesting that the taxonomic designation of the Mu1053 museum individual may be incorrect.

### Clade C (*C. crossleyi*)

Clade C is well supported in ML and Bayesian mtDNA gene trees and is unambiguously corroborated by all three nuclear markers. This group contains individuals identified as *C. crossleyi *in a previous mtDNA-based study [25] and also a single museum individual (Mu1050). In addition to the mtDNA-based identification of this group, morphometric analyses find similarity between the new field-sampled individuals and the Mu1050 museum *C. crossleyi *individual (LF Groeneveld, unpublished data).

It is important to note here a slight pattern of incongruence between the morphological and molecular results, with respect to *C. crossleyi *and *C. major*. Two newly sampled individuals, RMR146 and RMR164, are classified as *C. crossleyi *individuals according to all genetic data analyses, but cluster with *C. major *individuals in morphometric analyses. One of these individuals (RMR146) is sampled from a locality (25) that contains both species, suggesting that hybrid introgression may in part be responsible for such a pattern.

### Cheirogaleus minusculus

We have no molecular data to directly assess the status of *C. minusculus*. The single specimen upon which this species was described is listed in the museum catalogue of the British Museum of Natural History (NHM) as being from Ambositra/Antsirabe [45]. Our geographically closest sampling site to this locality is Ankazomivady (23), which is about 29 km south of the town of Ambositra. Morphometric and genetic analyses indicate these are *C. crossleyi *individuals, and they do not cluster with the *C. minusculus *individual in morphometric analyses. Thus, there is no indication that these individuals could represent *C. minusculus*. Unfortunately, the NHM does not allow sampling of holotype material for molecular analyses, which would be crucial to assess the status of this proposed species. A discussion within the museum community and a change in policy regarding invasive sampling of holotype material for molecular studies is needed to solve this dilemma for future studies.

### Genetic structure within the main *Cheirogaleus *lineages

Within the three species-level evolutionary lineages further genetic substructuring was detected via population genetic clustering. Genetic substructure was most tightly correlated with geography among populations within the clade A lineage, where some populations from the west and northwest (3, 4, 37) were placed in a distinct nuclear cluster, while populations from the northeast (7, 9) were placed in a second distinct cluster. These patterns indicate the potential for the further geographic isolation and divergence of populations beyond the three main *Cheirogaleus *lineages. However, the lack of concordant geographic patterns of divergence in the gene trees, especially in the mtDNA gene tree, indicate that this divergence may be very recent in nature. It is important to note, however, that Clade A is not entirely comprised of two genetic clusters, but also contains individuals that have cluster assignments more similar to individuals in the other two lineages. This same pattern is seen among individuals from Clades B and C, despite the fact that none of these individuals share alleles. It is possible that this pattern may result from an insufficient level of variation to accurately assign individuals. Additional molecular sampling in future studies is likely to be important in the adequate assessment of population-level structuring using genotypic clustering methods.

Our results do indicate, however, the special status of the Sambava locality; two species, *C. medius *and *C. crossleyi*, are found at this locality, and for both species the Sambava individuals are assigned to a genotypic cluster that largely separates them from other individuals of the respective species. This is most strongly seen in the case of *C. medius *and also includes the single sampled individual from the geographically close locality of Bekaraoka. Posterior probabilities for cluster assignments for some *C. crossleyi *Sambava individuals are low, but many individuals have high posterior probabilities (≥ 0.90) that clearly distinguish them from other populations of *C. crossleyi*. Again, these patterns appear to be signatures of more recent episodes of geographic isolation and divergence within the three main lineages of *Cheirogaleus*.

### Geographic distribution of the species

Results from the new sampling sites identified by this study allow us to further clarify the distribution of the three species of *Cheirogaleus *(Fig. 1). All three species are present in forest fragments in the Fort Dauphin region (southeastern Madagascar), as described by Hapke et al. [25]. The range of *C. medius *extends along the west coast up north to Ankarana (5). This species is, however, also found at two sites, Bekaraoka (7) and Sambava (9), on the northeastern coast. This is consistent with a presumed *C. medius *population at Daraina in northeastern Madagascar, as listed in Mittermeier et al. [46]. The distribution of *C. medius *is therefore not limited strictly to the western dry forests. The range of *C. crossleyi *extends from the southeastern tip of the island all the way north to Montagne d'Ambre (6) with many sampling sites being found inland along the eastern edge of the central plateau. There are a few exceptions in the north, both on the east and west coast: Sambava (9), Iharana/Vohemar (8), Ambanja/Beandroana (3), Manongarivo (1) and Ampijoroa (39). *Cheirogaleus major *is found, as previously described, in the eastern lowland forest, from the southeastern tip as far north as Maroantsetra (11). Additionally, there is one field (25) and one museum sampling site (22) along the eastern edge of the central plateau where *C. major *is found. We currently do not have any data to assess the populations mentioned in Thalmann and Rakotoarison [44], Ausilio and Raveloanrinoro [41], Thalmann [43] and Rasolofoson et al. [42].

### Multifaceted approach in species delimitation in lemurs

This study has shown that multiple independent lines of data can yield a robust estimate of species diversity. Morphological data alone are not expected to provide resolution of previously cryptic species, but indeed have had the opposite effect of over estimating species diversity [47]. The sole use of mtDNA might have diagnosed distinct populations, or even local matrilines as species and the use of any single nuclear marker would most likely lead to an underestimate of diversity. Only through combining all of these sources of data were we able to achieve a robust estimate of lineage diversity in *Cheirogaleus *and detect geographically structured patterns of genetic variation. Of course, it would be desirable to add more geographic and ecological information to the current data set, in order to verify and refine our conclusions. One starting point would be to examine the distinct populations identified through the population genetic cluster analysis, with special emphasis on the Sambava locality, where *C. medius *and *C. crossleyi *are found in sympatry. Furthermore, a direct comparison of the speciation patterns found in dwarf lemurs and those known for mouse lemurs, with estimates of time divergence dates, would be valuable to answer questions about the mechanisms driving this species radiation in Madagascar.

## Conclusion

Based on the general metapopulation lineage concept and multiple sources of data, we clarify the exclusivity of three of the seven recognized dwarf lemur species: *C. major*, *C. medius *and *C. crossleyi*. These three species were found to be genealogically exclusive in both mtDNA and nDNA loci, and furthermore, they exhibit morphological distinctiveness. Molecular and morphometric data support the hypothesis that *C. adipicaudatus *and *C. ravus *are synonymous with *C. medius *and *C. major*, respectively. *C. sibreei *falls into the mtDNA *C. medius *clade, but in morphological analyses the membership is not clearly resolved. We do not have sufficient data to assess the status of *C. minusculus*. Population genetic subdivisons are detected within these three species, but are not conclusive enough to warrant specific status.

The concordance-based approach, based on multiple independent lines of data, yielded a robust estimate of diversity within the genus *Cheirogaleus *and we conclude that this approach is well-suited for species delimitations. For dwarf lemur conservation this study implies that, since there are fewer species with greater individual abundances and distributions, dwarf lemurs should be less threatened than previously thought. Whether this implies that dwarf lemurs are not suitable flagship species and need not be regarded as intensely as other lemur taxa, or whether more emphasis in conservation should be placed on other measures than species numbers (e.g. as proposed by Sechrest et al., [3]) remains to be debated.

## Methods

### Sampling

Field samples from a total of 48 individuals across 14 localities in Madagascar were collected between March 2003 and May 2007 (Fig. 1, Table 1). Sampling sites were not evenly distributed across the island due to a bias in remaining forest cover [48]. Sampling was concentrated on the larger fragments found in the eastern portion of the island and makes no claim to be exhaustive. A maximum of three individuals per site, amounting to 31 individuals, were sacrificed and preserved as morphotypes and are now housed at the Département de Biologie Animale de l'Université d'Antananarivo, Madagascar. Tissue samples from internal organs (liver, kidney and spleen) and muscle tissue were stored in 70% EtOH. An additional 17 individuals were caught using Sherman live traps. One hundred traps were set along two to three transects for an average of 11 nights per site and baited with banana. Tissue for molecular analyses of these individuals was obtained by ear clipping after animals were anesthetized with GM2 [49]. External morphological measurements were taken from all 48 individuals, while internal measurements were only available for the 31 morphotypes. Animals were released at the site of capture at dusk on the following day. A total of 44 additional tissue samples were collected from specimens in three European museums: the Muséum National d'Histoire Naturelle, Paris (MNHN); Naturalis – Nationaal Natuurhistorisch Museum, Leiden, and the Natural History Museum, London (NHM). These specimens are the same individuals studies in the taxonomic revisions of Groves [24]. Small amounts of dried tissue were taken from skulls and in a few cases from skins. Of the 44 museum samples taken, we were able to include 17 in the final mtDNA analyses (Table 2). The presumed sampling sites of museum samples are marked with triangles on the map in Fig. 1. A total of 24 published *Cheirogaleus *haplotypes were incorporated into the analyses of the mtDNA data set (Table 3). Sequences from *Mirza zaza*, *Microcebus berthae*, *M. murinus*, and *M. ravelobensis*, which serve as representatives of other major cheirogaleid lineages [50], were used as outgroups to root the phylogenetic trees. All previously published GenBank sequences are listed in Table 3 and the sampling localities for *Cheirogaleus *sequences are marked with squares on the map in Fig. 1. All sequences generated for this study were deposited in GenBank under accession numbers EU825210-EU825610.

### Laboratory work

Genomic DNA was extracted from both field and museum tissue samples using the QIAamp™ DNA Mini Kit for DNA purification (QIAGEN) following standard protocol. For the field-collected samples two mitochondrial [cytochrome *b *(*cytb*), cytochrome oxidase II (*cox2*)] and three nuclear loci [adenosine receptor A3 exon 2 (*adora3*), alpha fibrinogen intron 4 (*fiba*), and von Willebrand Factor intron 11 (*vWF*)] were amplified, using primers and annealing temperatures given in Table 5. Amplifications were either carried out in 10 μl reactions containing a final concentration of 0.25 μM of each primer, 3 mM MgCl_2_, 0.25 mM dNTPs, 1× amplification buffer and 0.025 U/μl taq (Jumpstart, Sigma) or in 30 μl reactions containing a final concentration of 0.33 μM of each primer, 3 mM MgCl2, 0.166 mM dNTPs, 1× amplification buffer and 0.033 U/μl taq (Biotherm, Genecraft). Cycling conditions were 35 cycles of 30 s denaturation, 45 s annealing and 1 min. elongation except for two loci. The *adora3 *fragment only needed 45 s elongation due to its short fragment length and the two overlapping *cytb *fragments were amplified using 1 min. for all three steps. Amplification of the museum samples was carried out in 30 μl reactions containing a final concentration of 0.33 μM of each primer, 2 mM MgCl_2_, 0.166 mM dNTPs, 1× amplification buffer and 0.033 U/μl taq (Biotherm, Genecraft). Cycling conditions were 50 cycles of 1 min. denaturation, 1 min. annealing and 30 s elongation. All amplifications of museum samples were verified by replication in a second independent lab. Wax-mediated Hot Start PCR was used on all 30 μl reactions to increase specificity and yield. PCR products were purified employing Montage™ PCR Centrifugal Filter Devices (Millipore) according to manufacturer's instructions. For a little more than half of the nuclear sequences, due to multiple polymorphic sites in heterozygous individuals, PCR products were cloned into a pGEM vector (pGEM™-T EasyVector System I, Promega), averaging three clones per polymorphic sequence. Both strands of all PCR products were sequenced, using the respective primer pair used for amplification and standard vector primers M13F and M13R for the cloned products, employing the BigDye™ Terminator v1.1 Cycle Sequencing Kit (Applied Biosystems) on an ABI Prism™ 3100-Avant-Genetic Analyzer (Applied Biosystems).

**Table 5:** Primers used in this study

Locus	Primer	Primer sequence 5' - 3'	Reference	°C
*cytb*	L-CYT	AAT GAT ATG AAA AAC CAT CGT TGT A	[64]	55
	2763	GG(AG) ATT TT(AG) TCG GAG TCT GAT G	this study	
	2695	CCG ATT CTT CGC ATT CCA CTT	this study	55
	2510	GAC CAG (GT)GT ATT (AT)TT TAT ACT AC	C. Roos, pers. comm.	
	2877	ACG TAA AC(CT) ACG GCT GAA	this study	52
	2879	CCT CAG ATT CAT TCT ACT A	this study	
*cox2*	L7553	AAC CAT TTC ATA ACT TTG TCA A	[65]	48
	H8320*	CTC TTT AAT CTT TAA CTT AAA AG	[65]	
*adora3*	adora3F	ACC CCC ATG TTT GGC TGG AA	[66]	52
	adora3R	GAT AGG GTT CAT CAT GGA GTT	[67]	
*fiba*	Fiba-F	AAG CGC AAA GTC ATA GAA AAA G	[66]	56
	Fiba-R	CTA AAG CCC TAC TGC ATG ACC CT	[66]	
*vWF*	vWF-10	GAG CTG GAT GTC CTG GCC ATC CAT GGC AAC	[68]	60
	vWF-8	GAG TGC CTT GTC ACT GGT CAT CCC ACT TCA A	[68]	

The locus, primer name, primer sequence, reference and used annealing temperature in °C are given. The complete *cytb *locus was obtained by amplifying two overlapping fragments (primer pairs 1255, 2763 and 2695, 2510). The *cytb *fragments from museum material were amplified using primer pair 2877, 2879. *slightly modified from Adkins and Honeycutt(1994), fourth base not present in original primer.

### Phylogenetic analyses

Sequences were edited and aligned using Sequencher 4.7 (Gene Codes Corporation, Ann Arbor, MI, USA) and manually checked by eye. Subsequently, sequences were collapsed into haplotypes using MacClade 4.05 [51]. Haplotype data sets were used for all subsequent Maximum Likelihood (ML) and Bayesian phylogenetic analyses. For all mtDNA analyses *cytb *and *cox2 *fragments were concatenated, resulting in an alignment of 1824 bp. GenBank, museum and field sample sequences were collapsed into haplotypes separately, since, due to missing data, unambiguous assignment was not possible. Uncorrected "p" distances for the *cytb *locus were calculated as implemented in PAUP* v4.0b10 [52]. Optimal nucleotide substitution models for each locus were chosen using the Akaike Information Criterion (AIC) as implemented in Modeltest v3.7 [53]. All ML analyses were conducted using a genetic algorithm approach in Garli v0.951 [54]. In Garli only the model specifications settings were adjusted according to the respective data set; all other settings were left at their default value. Ten replicates were run for each data set to verify consistency in log likelihood (lnL) scores and tree topologies. Maximum-likelihood bootstrap percentages (BP) were estimated in Garli by performing 500 pseudoreplicate runs on each nuclear data set and 100 pseudoreplicates on the mtDNA data set. PAUP* v4.0b10 was then used to calculate a majority-rule consensus tree for each data set.

Bayesian analyses were conducted on a concatenated *cytb*+*cox2 *mtDNA data set and on the individual nuclear loci using MrBayes v3.1.2 [55,56]. For the mtDNA analyses, a partitioned analysis was performed treating the *cytb *and *cox2 *genes as separate partitions, each with their own DNA substitution models. In all analyses we used four Monte Carlo markov chains (MCMC) with the default temperature of 0.2. Analyses were run for ten million generations with tree and parameter sampling occurring every 100 generations. Flat priors were assumed for the model parameters including the proportion of invariable sites and the gamma shape parameter of rate variation among sites. The first 25% of samples were discarded as burnin, leaving 75,001 trees per run. The adequacy of this burnin and convergence of all parameters were assessed by examining the uncorrected potential scale reduction factor (PSRF) [57] as calculated by MrBayes v3.1.2 [55,56], which should approach 1 as runs converge and visual inspection of the trace of the parameters across generations using the software Tracer v1.3 [58]. Posterior probabilities (PP) for each split and a phylogram with mean branch lengths were calculated from the posterior density of trees using MrBayes v3.1.2 [55,56]. Phylogenetic trees were visualized using TreeEdit v1.0a10 [59] and FigTree v1.0 [60].

Statistical parsimony haplotype networks were constructed for each nuclear locus using the program TCS version 1.21 [61] for each individual nuclear locus. A 95% connection limit was used and gaps were treated as missing data.

### Population structure

A Bayesian population assignment test implemented in Structure v2.2 [62] was used to infer population structure based on a combined genotypic matrix from all four loci (*adora3*, *fiba*, *vWF*, and *mtDNA*) and also on the three locus nuclear matrix. An admixture model was used with correlated allele frequencies and no linkage among loci. For each number of populations assumed (K = 1 to K = 10) we performed 50 replicate runs. The four-locus data set was run with a burnin of 500,000 MCMC generations and 4 million subsequent generations. The three locus nuclear data set was run with a burnin of 250,000 generations and 1 million subsequent generations. The adequacy of the burnin and subsequent length of the MCMC chain was checked visually by plotting the parameters α and the lnL against the number of generations. In order to detect the favored number of genetic groups, an ad-hoc statistic ΔK [40] was calculated. In addition, a pairwise comparison of the 50 runs for each K was carried out using the perl script Simcoeff [63]. This procedure is based on the estimated membership fractions generated by Structure for a given K. The similarity coefficient for a pair of structure runs reflects the proportion of identical membership of individuals assigned through the Monte Carlo process. The proportion of runs resulting in 95% of the coefficients being equal is used to assess the stability of the cluster allocations.

The clustering pattern for the run with the highest probability (estimated log probability of the data) for K = 2 to K = 6 was visualized using Microsoft^® ^Excel^® ^v11.3.3. Membership coefficients with posterior probabilities under 0.05 were disregarded and proportionally added to the remaining membership coefficients. Therefore, in Fig. 6, some individuals show membership in fewer than K clusters.

## Authors' contributions

LFG carried out laboratory work, analyzed the data and wrote the paper. DWW analyzed the data and wrote the paper. RMR collected the samples. PMK and ADY conceived, financed and coordinated the study. All authors read and approved the final manuscript.

## Supplementary Material

Additional file 1 **vWF maximum likelihood phylogram.** ML phylogram based on an alignment of *vWF *haplotype sequences from 48 field samples. Tip labels contain the individual field numbers (E, RMR) of sequences within a haplotype. The sampling locality a haplotype was found in, are given in bold type in parentheses. ML bootstrap values and Bayesian posterior probabilities are depicted above the branches. Click here for file

Additional file 2 **fiba maximum likelihood phylogram.** ML phylogram based on an alignment of *fiba *haplotype sequences from 48 field samples. Tip labels contain the individual field numbers (E, RMR) of sequences within a haplotype. The sampling locality a haplotype was found in, are given in bold type in parentheses. ML bootstrap values and Bayesian posterior probabilities are depicted above the branches. Click here for file

Additional file 3 **adora3 maximum likelihood phylogram.** ML phylogram based on an alignment of *adora3 *haplotype sequences from 48 field samples. Tip labels contain the individual field numbers (E, RMR) of sequences within a haplotype. The sampling locality a haplotype was found in, are given in bold type in parentheses. ML bootstrap values and Bayesian posterior probabilities are depicted above the branches. Click here for file

Additional file 4 **Bayesian population structure analysis.** Bayesian assignment of the 48 field-collected individuals to populations assuming a population number of K = 2 to K = 6. Individuals are arrayed along the x-axis. The y-axis denotes the cumulative posterior probability of an individual's placement in particular population(s). Numbers in parentheses for each K indicate the number of identical solutions at a 95% threshold. Individuals are divided into populations by thin black lines. Populations are labeled at the bottom with numbers in parentheses corresponding to the sampling locality as marked in Fig. 1. (A) Results based on nuclear loci. (B) Results based on all loci. The solutions for each K, from K = 2 to K = 6, are consistent in that at each K + 1 the assignment of individuals to the clusters remains stable with just one cluster being split into two. There is only one exception to this pattern. The individual from Bekaraoka switches clusters from K = 2 to K = 3. Click here for file
