# Supplementary figures and images for: Species delimitation in lemurs: multiple genetic loci reveal low levels of species diversity in the genus Cheirogaleus
Source: BMC Evol Biol. 2009 Feb 4;9:30. doi: 10.1186/1471-2148-9-30 (PMC2652444; doi:10.1186/1471-2148-9-30)

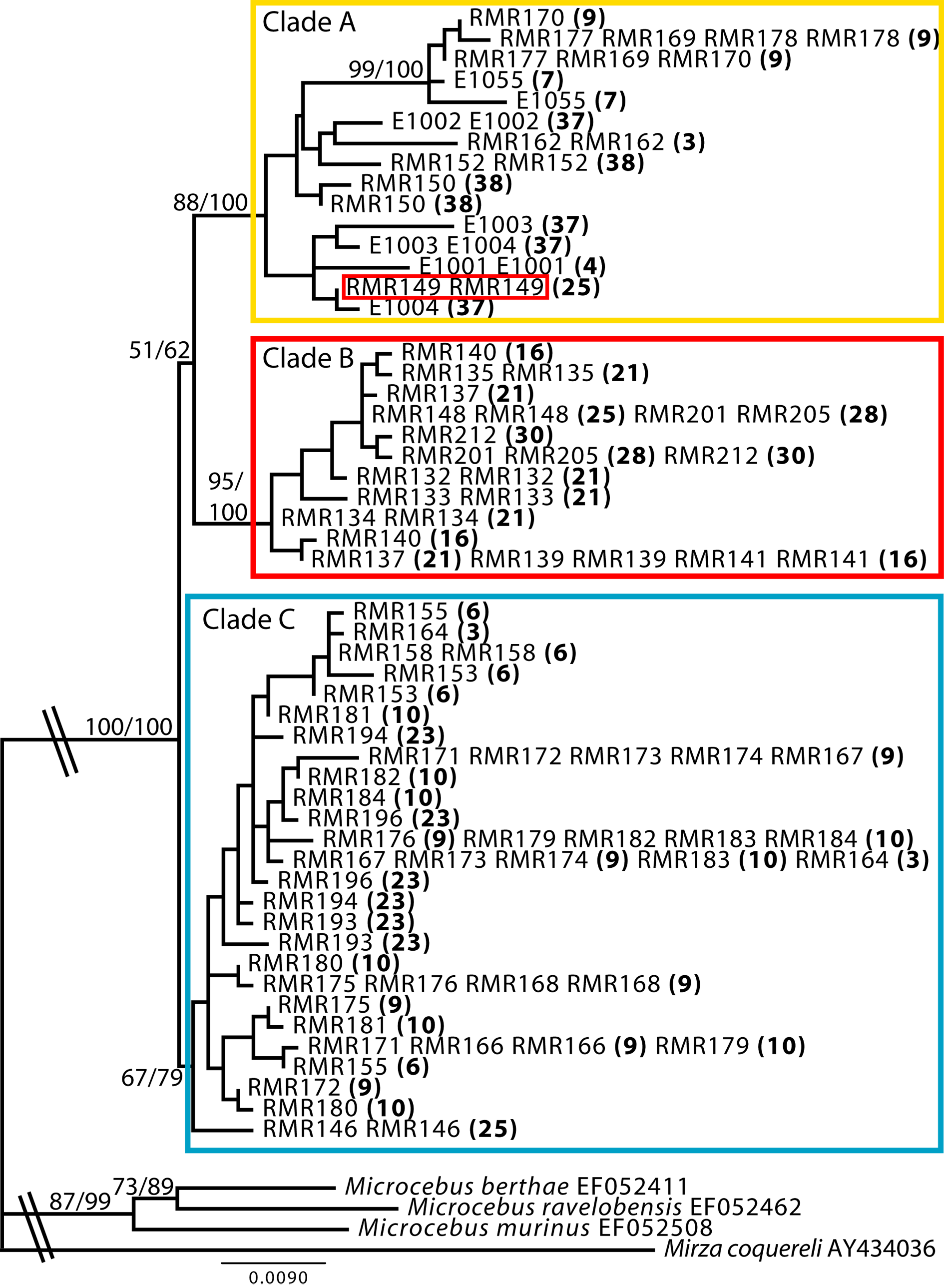

Supplement: Additional file 1 — vWF maximum likelihood phylogram. ML phylogram based on an alignment of vWF haplotype sequences from 48 field samples. Tip labels contain the individual field numbers (E, RMR) of sequences within a haplotype. The sampling locality a haplotype was found in, are given in bold type in parentheses. ML bootstrap values and Bayesian posterior probabilities are depicted above the branches. [file 1471-2148-9-30-S1.png]

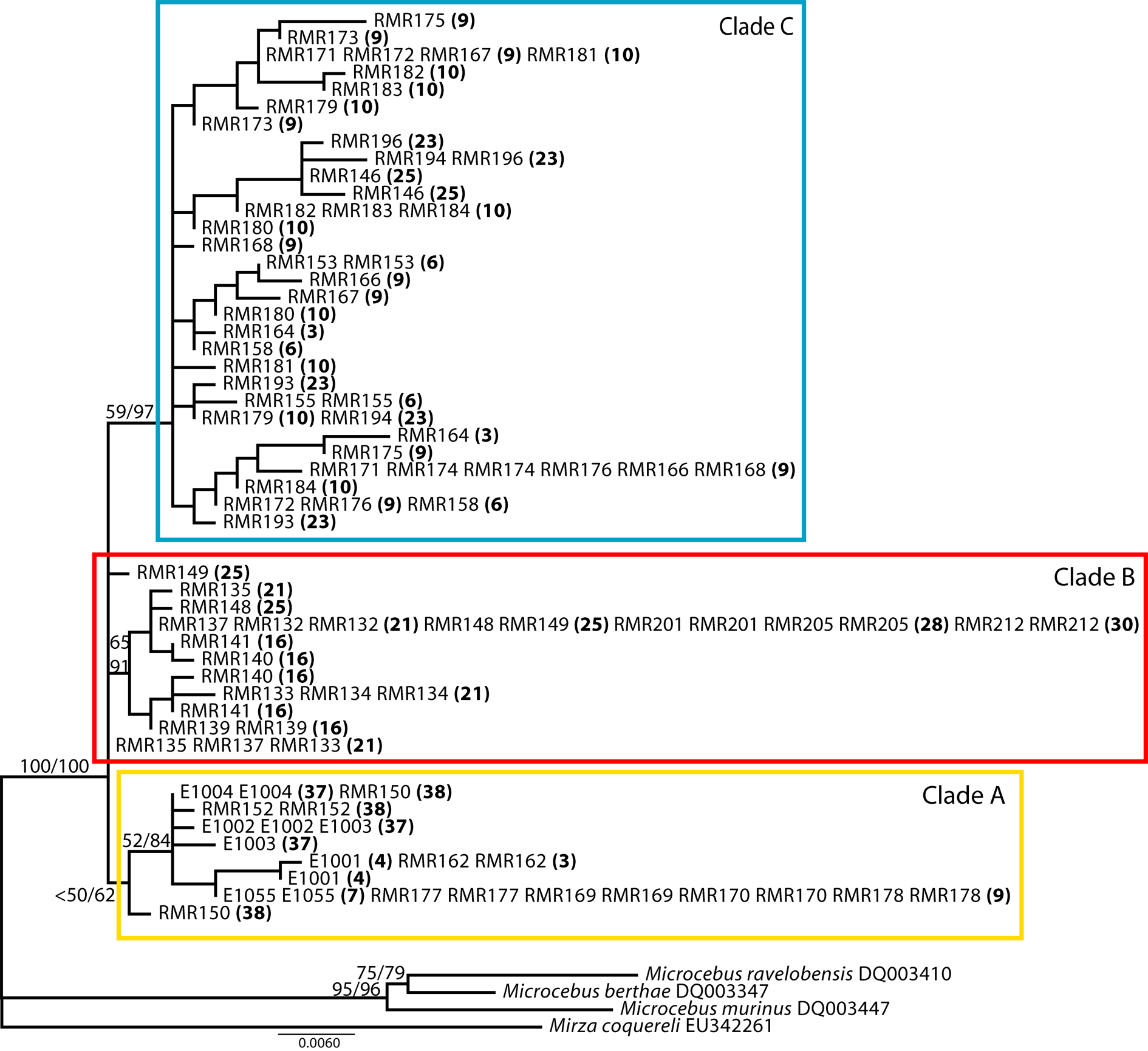

Supplement: Additional file 2 — fiba maximum likelihood phylogram. ML phylogram based on an alignment of fiba haplotype sequences from 48 field samples. Tip labels contain the individual field numbers (E, RMR) of sequences within a haplotype. The sampling locality a haplotype was found in, are given in bold type in parentheses. ML bootstrap values and Bayesian posterior probabilities are depicted above the branches. [file 1471-2148-9-30-S2.png]

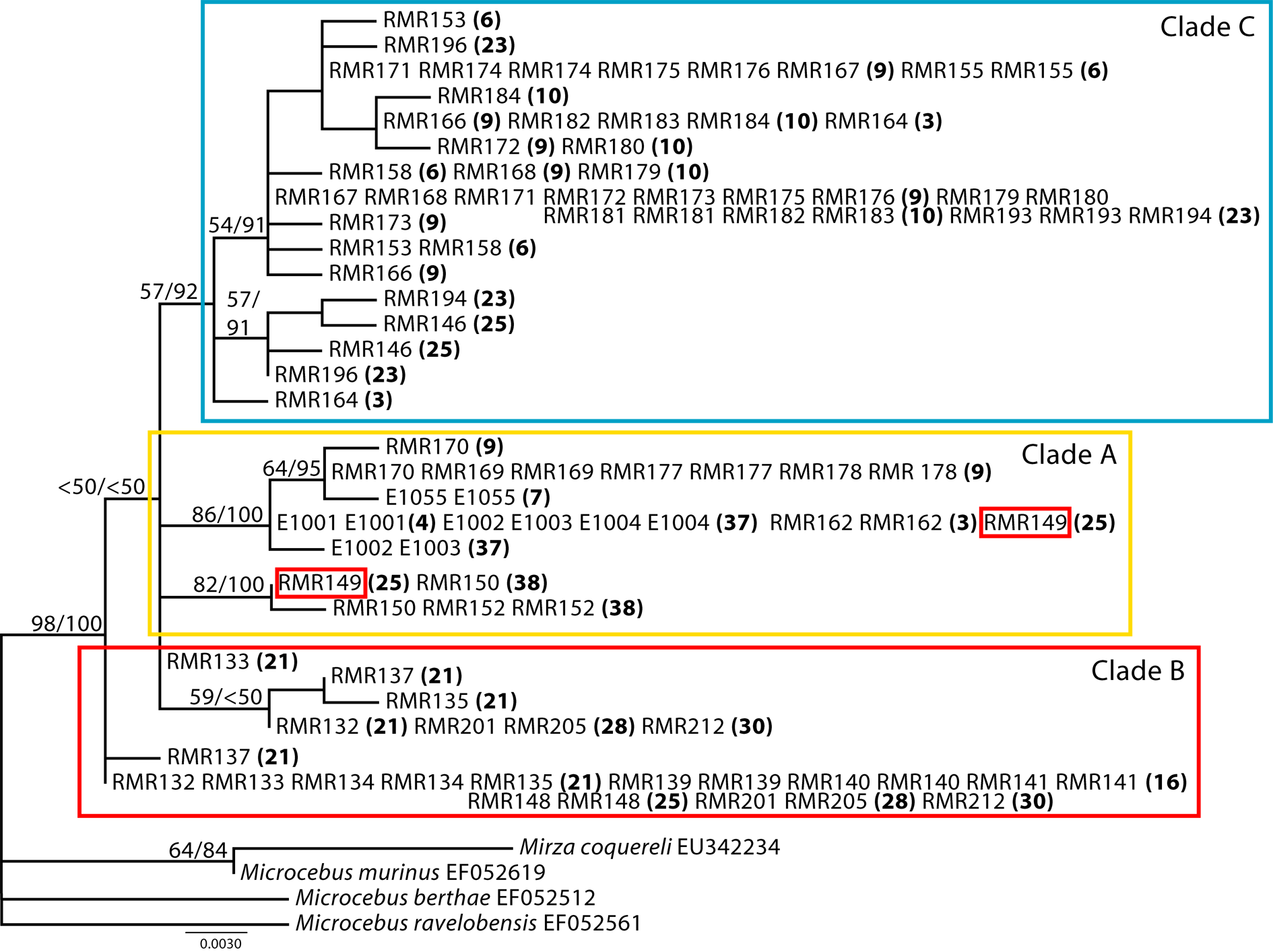

Supplement: Additional file 3 — adora3 maximum likelihood phylogram. ML phylogram based on an alignment of adora3 haplotype sequences from 48 field samples. Tip labels contain the individual field numbers (E, RMR) of sequences within a haplotype. The sampling locality a haplotype was found in, are given in bold type in parentheses. ML bootstrap values and Bayesian posterior probabilities are depicted above the branches. [file 1471-2148-9-30-S3.png]

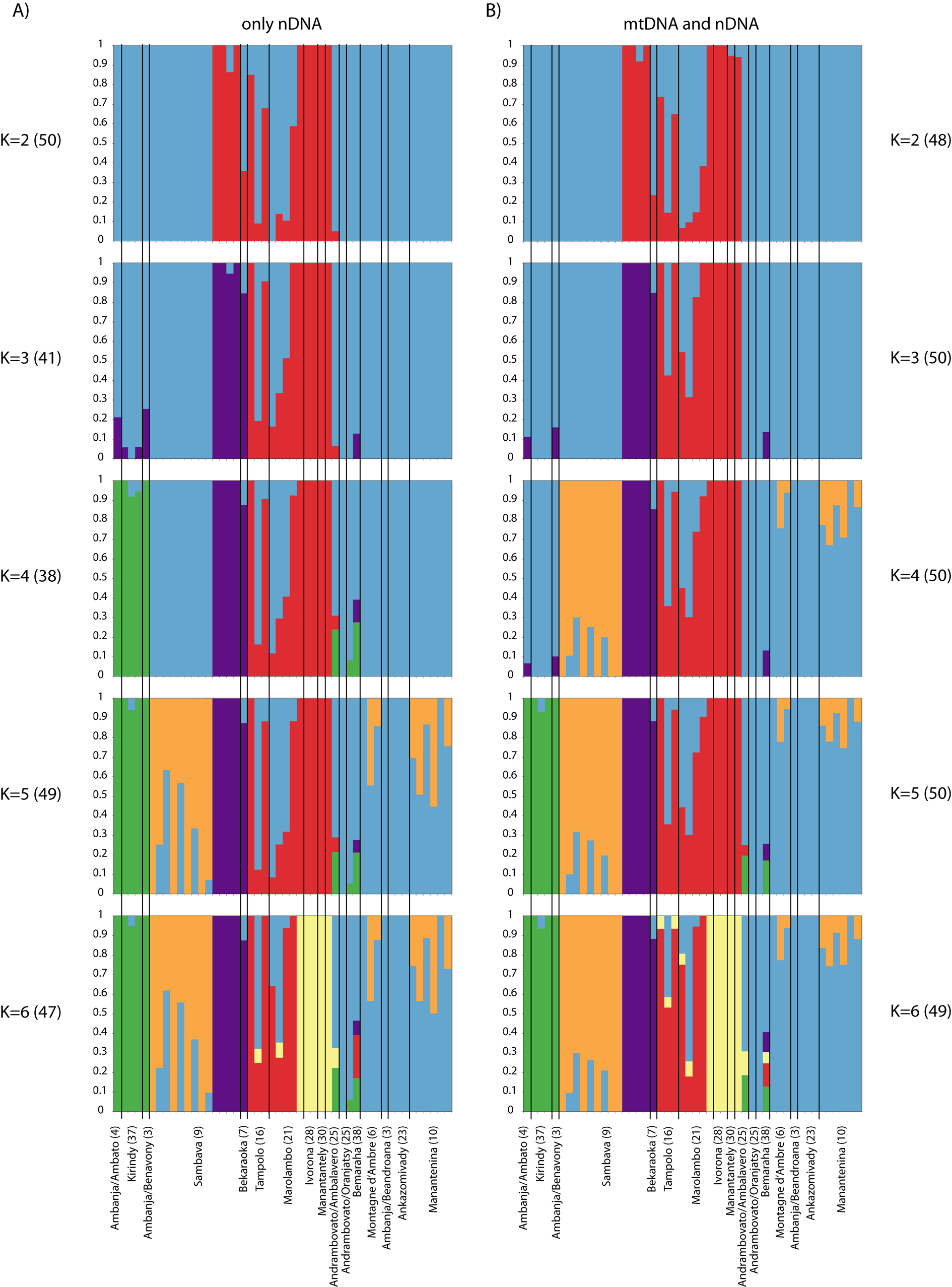

Supplement: Additional file 4 — Bayesian population structure analysis. Bayesian assignment of the 48 field-collected individuals to populations assuming a population number of K = 2 to K = 6. Individuals are arrayed along the x-axis. The y-axis denotes the cumulative posterior probability of an individual's placement in particular population(s). Numbers in parentheses for each K indicate the number of identical solutions at a 95% threshold. Individuals are divided into populations by thin black lines. Populations are labeled at the bottom with numbers in parentheses corresponding to the sampling locality as marked in Fig. 1. (A) Results based on nuclear loci. (B) Results based on all loci. The solutions for each K, from K = 2 to K = 6, are consistent in that at each K + 1 the assignment of individuals to the clusters remains stable with just one cluster being split into two. There is only one exception to this pattern. The individual from Bekaraoka switches clusters from K = 2 to K = 3. [file 1471-2148-9-30-S4.png]
